# Validity and reliability of the Turkish version of the Maternal Health Literacy Inventory in Pregnancy scale: a methodological study

**DOI:** 10.4069/whn.2024.10.18

**Published:** 2024-12-30

**Authors:** Yeşim Altuntas, Ayse Kilic Ucar

**Affiliations:** 1 Department of Obstetrics and Gynaecology, Istanbul Florence Nightingale Hospital, Istanbul, Turkiye; 2 Department of Nursing, Faculty of Health Sciences, Yeditepe University, Istanbul, Turkiye

**Keywords:** Health literacy, Literacy, Pregnancy, Pregnant women, Validity and reliability

## Abstract

**Purpose:**

This study aimed to translate the Maternal Health Literacy Inventory in Pregnancy (MHELIP) scale into Turkish and evaluate its validity and reliability for use in the Turkish population.

**Methods:**

The participants in this methodological study included 250 pregnant women who presented to the antenatal clinic of the Florence Nightingale Hospital in Istanbul, Turkiye. Content validity was assessed using expert approval. Confirmatory factor analysis, exploratory factor analysis, and structural equation modeling were used to assess the validity. Criterion validity was evaluated using the short-form health literacy survey tool, the Short-Form Health Literacy Questionnaire (HLS-SF12). To assess reliability, Cronbach’s alpha, item analysis, and the test-retest method were used.

**Results:**

The mean age of the participants was 32.02±4.15 years. The content validity index of the scale was .99. The scale had a four-factor structure that fit well with 48 items. “Maternal health knowledge,” “maternal health information search,” “maternal health information assessment,” and “maternal health decision making and behavior” subscales had Cronbach’s alpha values of .91, .76, .85, and .90, respectively. The MHELIP and HLS-SF12 scores were significantly correlated (*r*=.422, *p*<.001).

**Conclusion:**

The MHELIP was found to be a valid and reliable measurement tool in pregnant Turkish women.

## Introduction

Pregnancy is one of the most sensitive and important periods in a woman’s life. The health and well-being of the mother directly affect the life of the fetus during this period [1]. Initiating prenatal care during the early stages of pregnancy will lead to early detection of most related complications and the prevention of problems that may occur in the mother and the infant, therefore improving the pregnancy and birth outcomes [2,3]. However, certain factors can prevent proper and timely care during pregnancy. One of these factors is an insufficient level of maternal health literacy (MHL) during pregnancy [3-5].

MHL refers to the skills (motivational, cognitive, and social) needed by a pregnant woman to obtain, understand, and apply the knowledge that she needs to protect and advance the health of herself and her baby [3]. In addition, MHL includes the skills to recognize dangerous symptoms during the pregnancy period, manage a healthy lifestyle and proper nutrition, focus on the realities of pregnancy, and learn birth and baby care skills [6]. Adequate MHL is essential for a pregnant woman so that she can effectively monitor her own health and make correct decisions regarding the baby’s health [4,5]. Studies have shown that high MHL results in better maternal and child health behaviors and outcomes, such as efforts to obtain health information, more effective use of prenatal care services, more effective and longer breastfeeding, efforts to find appropriate solutions to health problems, and decreased health-related expenditures [4-8].

Pregnant women with low MHL experience unplanned pregnancies more often, do not have timely prenatal tests performed, miss their appointments more often, experience postpartum depression more frequently, take fewer vitamin supplements, use more drugs, have difficulty managing the pregnancy period, have babies with lower birthweight and Apgar scores, experience more birth trauma, and are more likely to stop breastfeeding and using maternal milk in the first 6 months after birth [4-11].

The level of health literacy falls into four categories based on the index values obtained: 0–25, inadequate/poor; >25–33, problematic-limited health literacy/poor; >33–42, adequate health literacy/good; and >42–50, excellent health literacy [4,12]. Health literacy levels vary across countries. Some studies have reported the prevalence of MHL to vary between 10% and 45.5% [4,8-10]. According to the European Health Literacy Survey (2015), Bulgaria had the highest rate of inadequate health literacy (27%), while the Netherlands had the highest rate of excellent general health literacy (25%) [4]. A study from Iran found that only 18% to 24% of pregnant women had good health literacy, and 31% to 34% had poor health literacy [11]. The rates for inadequate, problematic, sufficient, and excellent health literacy rates in Turkiye have been reported to be 24.5%, 40.1%, 27.8%, and 7.6%, respectively [12].

A review of the MHL-related studies in Turkiye showed that MHL levels closely parallel general health literacy levels. Other studies revealed that approximately one-third of pregnant women have “inadequate or limited” health literacy levels [13-15]. Pregnant women with low MHL levels have been shown to have a poor understanding of prenatal care, use prenatal services less, gain less weight during pregnancy, pay less attention to their nutrition, know less about the danger signs during pregnancy, rely more on traditional health practices, and need more help during the birth and postnatal baby care process [13-16]. These results of low MHL significantly increase healthcare and treatment costs, negatively impacting the healthcare system in Turkiye.

Identifying current MHL levels is the first and most important step in increasing those levels and improving maternal and infant health. Increasing MHL will increase the patient’s efficacy in understanding and using health-related information and their ability to benefit from healthcare services. Increasing the healthcare literacy of pregnant women can be achieved by developing communication between healthcare professionals and these women. Providing healthcare training to pregnant women with low MHL levels will enable them to use and benefit from healthcare services, thus protecting the health of the mother and baby at the highest level [13-16]. The objectives of healthcare services should include determining and increasing the healthcare literacy of pregnant women.

There are several tools that measure general health knowledge [12,15,17-19]. However, using group or situation-specific scales, instead of those that measure general health information during pregnancy, will provide more valid and reliable results. There was no Turkish MHL scale at the time this study was started. The aim of the current study was to translate the Maternal Health Literacy Inventory in Pregnancy (MHELIP) scale [3], which is used to measure pregnant women’s MHL levels, and to determine its validity and reliability.

## Methods

**Ethics statement**

The study was approved by the Ethics Committee of Demiroglu Science University (17.01.2023/2023-02-07) and Istanbul Florence Nightingale Hospital (27.12.2022, IDR.2022-1083). Participation was voluntary and informed consent was obtained from all participants.

### Study design

This was a methodological study to determine the validity and reliability of a Turkish translation of the MHELIP. The STROBE (STrengthening the Reporting of OBservational studies in Epidemiology) guidelines [20] were used in the reporting of this study.

### Sample and sampling

Inclusion criteria for this study included pregnant women who (1) were aged >20 years old, (2) had not received a medical education, (3) were not a health care worker, (4) were literate in Turkish, and (5) volunteered for the study. Those who did not fully complete the data collection form were excluded. The scale development guidelines of DeVellis and Thorpe [21] suggest that a validity and reliability study conducted to adapt a scale to another culture should reach at least 5 to 10 times as many participants as the number of scale items [21]. Since the MHELIP consisted of 48 items, we aimed for 250 pregnant women who visited Istanbul Florence Nightingale Hospital for routine pregnancy care.

### Measurement

Data collection took 15 to 20 minutes. The questionnaire was piloted among 20 pregnant women who were recruited separately and not included in the main study.

The MHELIP developed by Taheri et al. [3] in Iran was translated according to guidelines for the intercultural scale adaptation stages for language and culture adaptation, as updated by Çapık and Gözüm [22]. We used the translation-reverse translation method, in accordance with these guidelines to adapt the measurement tool into Turkish, the target culture’s language. The adaptation of the scale to Turkish was performed in three stages. In the first stage, three independent persons who were specialists in nursing, knew English well, and whose native language was Turkish translated the original scale from English into Turkish. In the second stage, each translated Turkish item was checked for meaning and grammar and a single form was created. In the third stage, the Turkish scales were translated into English by two persons who were specialists in nursing, knew Turkish well, and whose native language was English. The scale was then checked again for English meaning and grammar. There was no need to make any changes to the questions.

The MHELIP contains 48 items and four subscales. The tool is rated on a 5-point Likert-type scale from 1 (I don’t know it at all) to 5 (I know it fully). Subscale dimensions include: (1) maternal health knowledge (MHK, items 1–21); (2) maternal health information search (MHIS, items 22–27); (3) maternal health information assessment (MHIA, items 28–33); and (4) maternal health decision making and behavior (MHDMB, items 34–48). Higher scores indicate higher MHL levels.

To calculate the score for each subscale or the total score, the raw scores are added first and linearly converted into a score between 0 and 100 using the following formula: score=(raw score–minimum possible raw score)/(maximum possible raw score–minimum possible raw score)×100.

The MHELIP score is interpreted as inadequate (≤50.0), problematic (50.1–66.0), adequate (66.1–84.0), or excellent (>84.1) [3]. While the inadequate and problematic categories define limited health literacy, the adequate and excellent categories define desired health literacy [3].

In the original study [3], Cronbach’s alpha was .94 (total), and the values for the subscales were .94 for MHK, .66 for MHIS, .79 for MHIA, and .87 for MHDMB. In this study, Cronbach’s alpha for the total scale was .94, and the values for the subscales were .91 for MHK, .76 for MHIS, .85 for MHIA, and .90 for MHDMB. Authorization was received from the authors of the original study to use the MHELIP.

The Short-Form Health Literacy Questionnaire (HLS-SF12) is a 12-item scale, originally developed to determine health literacy levels in the general public [23] and can also be used in studies of women [24]. The scale has high reliability with a Cronbach’s alpha of .85 and includes 4-point Likert-type response options ranging from 1 (very difficult) to 4 (very easy). It consists of 12 items, categorized into three criteria: good (score >36), moderate (score 28–35), and poor (score <28) [23]. The Turkish HLS-SF12 showed factor loadings of .47 to .70, item total correlations of .39 to .61, and a Cronbach’s alpha reliability coefficient of .85 [18]. Permission to use the HLS-SF12 was obtained from the developers (Karahan Yılmaz and Eskici) [18]. Finally, to assess the sociodemographic and pregnancy-related characteristics of the participants, the researchers put together 13 items based on the literature [3,4,11,12].

### Study procedure

This methodological study was conducted between February and May 2023 at the antenatal clinics of Istanbul Florence Nightingale Hospital in Turkiye. The data were collected by the researcher in face-to-face interviews with women who had agreed to participate voluntarily (no payment made). After the purpose of the study was explained, they were asked to fill in data forms, and their responses were collected upon completion.

To evaluate the validity of results, content validity, construct validity, and criterion validity tests were used. To evaluate reliability, Cronbach’s alpha, item analysis, and test-retest analyses were used.

### Validity

#### Content validity

The Davis technique was employed to determine content validity [22,25]. Ten nurses who were experts in gynecology and obstetrics were asked to test the content validity of the Turkish version of the MHELIP scale (MHELIP-TR). The experts were asked to grade each MHELIP-TR item on a scale of 1 to 4 (1, not suitable; 2, partially suitable but needs revision; 3, partially suitable but needs minor revision; and 4, very suitable). Afterwards, the scale items were checked by the researchers and the final scale items were determined. The lowest acceptable content validity index (CVI) value was set at 0.80 [22,25].

#### Construct validity

Confirmatory factor analysis (CFA) and path analysis were used to evaluate the construct validity of the MHELIP-TR. Before applying factor analysis, the conformity of the data taken from the sample for analysis and the adequacy of the data set and sample size were evaluated using the Kaiser-Meyer-Olkin (KMO) test. Bartlett’s test of sphericity was used to evaluate whether the data came from a multivariate normal distribution. If the KMO was higher than .60 and Bartlett’s test of sphericity was significant, the data were fit for factor analysis [22].

A comparative fit index was used in the CFA and path analysis for construct validity. The model was accepted if the data obtained was within the appropriate reference ranges. If not, recommendations in the modification indices were followed to improve the fit indicators of the model [26].

#### Criterion validity (equivalent form reliability/scale validity)

Criterion-based validity is confirmed when the calculated correlation coefficient is statistically significant. Positive, high, and significant correlations between the scales at the end of these analyses prove that the new scale measures the feature it is trying to measure. The HLS-SF12 was used to determine equivalent form validity. The relationships between two independent numerical variables were analyzed using the Pearson correlation coefficient [27].

### Reliability

Cronbach’s alpha, item analysis, and test-retest analyses were used to assess the reliability of the MHELIP-TR.

Cronbach’s alpha was measured to assess the reliability of the MHELIP-TR and its subscales. A Cronbach’s alpha closer to 1.0 (range, 0–1) indicated higher reliability (excellent, .93–.94; strong, .91–.92; and reliable, .84–.90) [21,25].

Item analyses were performed to evaluate the contribution of the scale items to the scale. When evaluating total correlation, the items with a correlation of .30 and above indicated that the item was distinctive [27].

For test-retest reliability, it is recommended that the instrument be applied twice within 2 to 4 weeks on at least 30 samples [22,28]. For the test-retest in this study, a sample of 50 pregnant women were invited from the 250 sample. Reliability coefficients closer to +1 indicated that the reliability was high [26,27]. The external consistency (resistance to time) coefficient was calculated with test-retest.

### Statistical analysis

IBM SPSS version 22.0 software (IBM Corp., Armonk, NY, USA) was used for statistical analysis of the data. Number, percentage, mean, and standard deviation were used for descriptive statistics. Other psychometric analyses are as described above.

## Results

### Characteristics of the study population

The mean age of the 250 participants was 32.02±4.15 years (range, 20–41 years). Most had a university degree or higher (86.0%). Approximately three-quarters were working in paid employment (74.8%), and half (51.2%) perceived their income status as “income equal to expenses.” The mean number of pregnancies was 1.44±0.77 and 69.6% of the participants were nulliparous (Table 1).

The mean MHELIP-TR score was 73.77 (range, 0–100), indicating adequate or desired health literacy. The mean scores of the subscales were: MHK 68.44 (adequate or desired health literacy), MHIS 62.68 (problematic or limited health literacy), MHIA 77.55 (adequate or desired health literacy), and MHDMB 84.17 (excellent or desired health literacy) (Table 2).

### Validity results

#### Content validity

No item had a CVI below .80 in the current study, and the mean CVI of the total items in the scale was .99.

#### Construct validity

The results of the KMO test (.89) and Bartlett sphericity test (B=7857.79, *p*<.001) showed that the data were suitable for analysis. Based on this information, the main application phase started with 48 items.

CFA was used to test whether the MHELIP-TR formed a holistic structure and found that it was a statistically appropriate model with goodness of fit indicators that were within the reference ranges specified in the literature. Although the chi-square value was expected to be insignificant in the CFA, it has been found to be significant in cases where the sample size is >250 [23]. Therefore, it was possible to explain the significance of the value in question based on the largeness of the number (Table 3).

#### Path diagram

**Figure 1.**
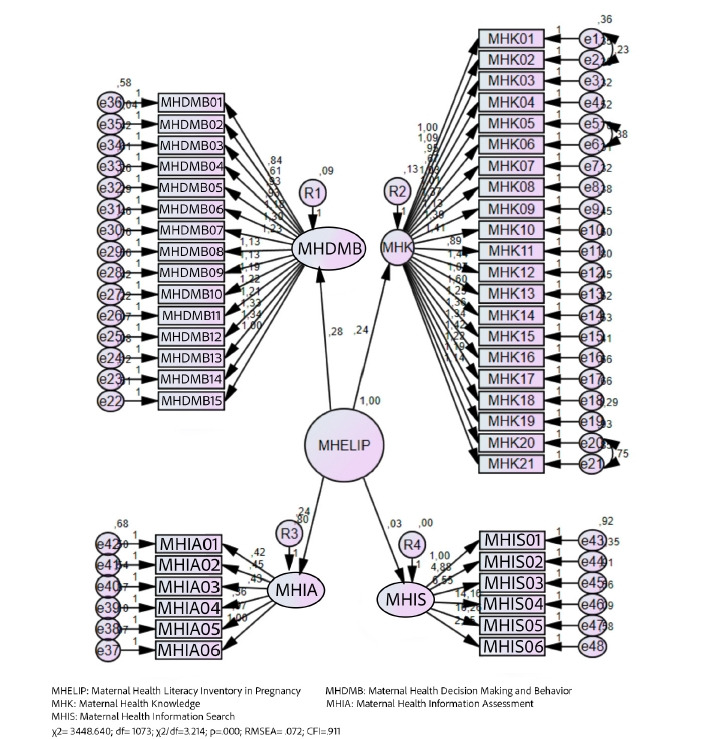
Path diagram.

Using CFA, we also found the variance and covariance values of the MHELIP-TR scale to be within an acceptable range and to be statistically significant (*p*<.001). The CFA conducted to identify whether the factors of the MHELIP-TR formed a holistic structure showed the four factors in the scale to be within the same structure, confirming the factor construct of the scale (Figure 1).

#### Criterion validity

The relationship between the MHELIP-TR and the HSL-SF12 was found to be significantly correlated with moderate strength (*r*=.422, *p*<.005). The relationships with the total score of the scale were also found to be positively significant.

### Reliability results

#### Cronbach’s alpha

The Cronbach’s alpha of the MHELIP-TR was .94, with the subscales between .70 and .91. These values revealed extremely high reliability and internal consistency (Table 2).

Item and factor differences between the lower 25% and upper 25% quartile groups of the MHELIP-TR were evaluated with the independent samples t-test. Differences between the mean group values were found to be significant (*p*<.001). Therefore, all items and factors of the measurement tool were capable of distinguishing individuals with and without the measured characteristic.

#### Item analysis based on item total correlation

To evaluate the contribution of scale items to the scale, Pearson correlation analysis was conducted to identify the relationships between the MHELIP-TR items and the total scale scores. This revealed significant positive correlations between all items and the total scale scores (*p*<.001).

In addition, positive and significant relationships were found between all factors of the MHELIP-TR. Although the correlation values of the different factors varied (*p*<.001), the factor scores showed equally high correlations with the overall total scale scores (*p*<.001).

#### Test-retest results

Analysis revealed a high positive correlation between the measurements (*p*<.001) after a 15-day interval. The correlation was also significantly positive for the total scale score (*r*=.732, *p*<.001) (Table 4). According to the construct validity and reliability item analyses of the MHELIP-TR scale, the 48-item, four-factor scale construct was preserved, and the construct was verified.

## Discussion

For research to yield healthy results, the validity and reliability of the scales used in the research are important factors. For this reason, we felt it was useful to explain in this paper how the reliability and validity of the scale were measured. Our study showed that our Turkish version of the MHELIP was a valid and reliable tool for use with pregnant women.

At the time of this study, there was no Turkish version of the MHELIP available to our knowledge. Thus, this study was conducted with permission from the developers Taheri et al. [3]. After data collection was completed for this study, the study by Abay et al. [29] was published, which translated the MHELIP and performed psychometric testing with data from 2021 to 2022. In this point, our work results were evaluated using the findings of both Taheri et al. [3] and Abay et al. [29]. After translation-reverse translation of the scale, content validity, construct validity, criterion validity, internal reliability, and test-retest reliability were established.

In this study, participants were slightly older (32.02±4.15 years) than those in the studies by Taheri et al. [3] (28.32±5.59 years) and Abay et al. [29] (29.24±5.77 years). In addition, the educational status of the women in this study (86.0% university and higher education) was higher than in the studies by Taheri et al. [3] (41.6% high school) and Abay et al. [29] (42.2% high school). When considering pregnancy status, 69.6% of the women in this study, 51.3% in Taheri et al.’s study [3], and 32.2% in Abay et al.’s study [29] were nulliparous. The rates of mean age, education level, and first pregnancy status were higher in our study, likely due to the fact that the hospital where our study was conducted was a private hospital. Our findings demonstrated that the MHELIP-TR scale can be used as a valid and reliable measurement tool to determine the level of health literacy in pregnant women of different ages, educational status, and number of pregnancies.

Content validity is the extent to which a measurement tool encompasses the content it is supposed to measure. Ten expert opinions were obtained in this study. The CVI score of all expressions in the scale was 0.99; therefore, no items were removed. Our results showed that the Turkish version of the MHELIP demonstrated construct validity. Taheri et al. [3], who developed the original scale, consulted 14 experts and obtained scope validity after extracting an item with a KMO value lower than .51. Abay et al. [29] used 16 experts in their study, and their scale had content validity ratios and a CVI >0.50; therefore, no items were removed.

A holistic scale should cover all dimensions of the concept [3]. In this study, the KMO value was .89 and the Bartlett value was B=7,857.79 (*p*<.001). These findings demonstrate the construct validity of the MHELIP-TR scale. The MHELIP items in the study by Abay et al. [29] had factor loadings of 0.57 to 0.92, also demonstrating construct validity. As for criterion-related validity, both this study and Abay et al.’s study [29] found significant correlation coefficients of moderate strength. In this study, the relationship between the MHELIP-TR and HSL-SF12 (*r*=.42, *p*<.005) was comparable to the correlation between the MHELIP and health literacy index (HLI) used in Abay’s study (*r*=.57, *p*<.001) [29]. In both studies, the higher the MHELIP score, the higher the HLS-SF12 and HLI scores, indicating that the Turkish version of the MHELIP was a reliable measurement tool. Given that the goodness of fit indicators was found to be at the levels of “good fit” and “excellent fit,” the MHELIP-TR was approved and the structure validity of the scale was ensured.

The Cronbach’s alpha of the MHELIP-TR was .94 in this study, which indicated excellent internal consistency. It was comparable to the Cronbach’s alpha values of Taheri et al.’s original scale (.94) [3] and Abay et al.’s version (.96) [29]. The subscales also had acceptable and desirable Cronbach’s alpha values, suggesting that the MHELIP-TR was a consistent measurement tool.

The original developer used the test-retest reliability method with a 2-week interval and reported appropriate stability for the scale (intraclass correlation coefficient=.96) [3]. Our study also included test-retest reliability, while it was not performed in Abay et al.’s study [29]. The analysis results revealed a high positive correlation between the measurements (*p*<.001) after a 15-day interval. The correlation was also found to be significantly positive for the total scale score (*r*=.73, *p*<.001).

The study had some limitations. First, the study was conducted at one private hospital based in the capital city of Turkiye. The pregnant women included in this study were highly educated and above the national average for 2022 (46.2% with primary and secondary education, 21.6% with university level or higher) [30]. Thus, the level of MHL may not be applicable to other samples of pregnant women and requires further study. Another limitation was that most of the pregnant women in our study were primiparous (69.6%); thus, the findings may not directly apply to nulliparous women.

In conclusion, the Turkish version of the MHELIP (consisting of 48 questions and four subdimensions) is a valid and reliable measure of MHL during pregnancy. Thus, health professionals can use this scale to identify MHL levels in the Turkish population. It can also contribute to helping patients identify and understand healthcare information, learn to use this information, and benefit from healthcare services. More studies, with larger samples of pregnant women, are needed to further evaluate health literacy using the MHELIP-TR scale.

## Figures and Tables

**Table 1.** Descriptive characteristics of the participants (N=250)

Variable	n (%) or mean±SD
Age (yr)	32.02±4.15
Education level	
Primary and secondary school	9 (3.6)
High school	26 (10.4)
University degree and above	215 (86.0)
Employment status	
Yes	187 (74.8)
No	63 (25.2)
Income status	
Income less than expenses	33 (13.2)
Income equal to expenses	128 (51.2)
Income more than expenses	89 (35.6)
Number of pregnancies	
Nulliparous	174 (69.6)
Multiparous	76 (30.4)
Total number of pregnancies	1.44±.77
Presence of chronic disease	
No	212 (84.8)
Yes	38 (15.2)

**Table 2.** Distribution of the MHELIP-TR and subscale scores (N = 250)

MHELIP-TR and subscales	Item	Score (0–100)	Cronbach’s alpha
Overall MHELIP-TR	48	73.77	.94
Maternal Health Knowledge	21	68.44	.91
Maternal Health Information Search	6	62.68	.76
Maternal Health Information Assessment	6	77.55	.85
Maternal Health Decision Making and Behavior	15	84.17	.90

MHELIP-TR: Turkish version of the Maternal Health Literacy Inventory in Pregnancy.

**Table 3.** Goodness of fit for the measurement indicators in the MHELIP-TR using confirmatory factor analysis

Measurement indicator	Excellent fit	Good fit	Scale values	Fit degree
χ^2^			3,448,64	
*p*-value	≥.001, ≤.005	≥.001, ≤.005	.002	Excellent fit
df			1,073	
χ^2^/df	≥0/≤3.00	≥3/≤5.00	3.214	Good fit
RMSEA	≥0, ≤.05	≥.05, ≤.08	.07	Good fit
CFI	≥.95, ≤1.00	≥.90, ≤.95	.91	Good fit
AGFI	≥.90, ≤1.00	≥.85, ≤.90	.93	Excellent fit
TLI	≥.95, ≤1.00	≥.90, ≤.95	.002	Excellent fit

MHELIP-TR: Turkish version of the Maternal Health Literacy Inventory in Pregnancy.

AGFI: Adjusted Goodness of Fit Index; CFI: Comparative Fit Index; df: degree of freedom; RMSEA: Root mean square error of approximation; TLI: Tucker-Lewis Index.

**Table 4.** MHELIP-TR test-retest correlation analysis

Subdimension	n	*r*	*p*
MHELIP-TR, total score	50	.73	<.001
Maternal Health Knowledge	50	.84	<.001
Maternal Health Information Search	50	.77	<.001
Maternal Health Information Assessment	50	.50	<.001
Maternal Health Decision Making and Behavior	50	.65	<.001

MHELIP-TR, Turkish version of the Maternal Health Literacy Inventory in Pregnancy.
